# Complete Genome Sequence of Mycobacterium bovis BCG SL222 Sofia, the First WHO Reference Reagent for the M. bovis BCG Vaccine of the Russian BCG-I Substrain

**DOI:** 10.1128/MRA.01318-20

**Published:** 2021-01-21

**Authors:** Stefan Panaiotov, Yordan Hodzhev, Vladimir Tolchkov, Alexander Mihailov, Roumen Kofinov, Todor Kantardjiev, Tzvetelina Stefanova

**Affiliations:** a National Center of Infectious and Parasitic Diseases, Sofia, Bulgaria; b BB-NCIPD Ltd., Sofia, Bulgaria; University of Maryland School of Medicine

## Abstract

Mycobacterium bovis BCG SL222 Sofia is a substrain descending from the Russian BCG-I vaccine strain. Here, we report the complete genome sequence of BCG SL222 Sofia, which will facilitate identity assurance and will contribute to more consistent manufacturing, standardization, and differentiation of substrains used in vaccine production.

## ANNOUNCEMENT

Currently, the main substrains used for BCG vaccine production are Danish (Copenhagen-1331), Japanese (Tokyo-172-1), Brazilian (Moreau/Rio de Janeiro), Russian (Moscow-368), and Bulgarian (SL222 Sofia) (1–3). Historical records showed that BCG SL222 Sofia is derived from generation 374а of BCG-I (Russia). The BCG-I generation 374а lyophilized batch was produced in February 1971 in Moscow, and samples of it were sent to Bulgaria. The Russian strain was received from the Pasteur Institute in Paris in 1925 and designated BCG-I (4, 5).

Primary seed lot SL222 Sofia was produced in May 1972 directly from generation 374a of BCG-I, according to the WHO requirements for dried BCG vaccine (6, 7), at the Laboratory for BCG Vaccine, National Centre of Infectious and Parasitic Diseases (Sofia, Bulgaria). Microbial growth was performed on glycerinated potato medium (8) and Sauton’s medium (9). The freeze-dried BCG SL222 Sofia was verified as Mycobacterium bovis BCG (6, 7).

BCG SL222 Sofia was cultured on Löwenstein-Jensen medium for 35 days at 37°C. The DNA was isolated according to the procedure described by van Soolingen et al. (10), including incubations with lysozyme, 10% SDS, proteinase K, and 10% cetyltrimethylammonium bromide (CTAB), protein/lipid extraction with phenol-chloroform, and DNA precipitation with isopropanol.

We combined Illumina and Nanopore approaches to assemble the genome sequence of BCG SL222 Sofia. The same DNA preparation was used for both libraries. An Illumina library was prepared with the Nextera DNA Flex kit and Nextera DNA CD indexes, and 2 × 300-bp sequencing was performed on a MiSeq sequencer using the MiSeq reagent kit v.3 (Illumina, USA). Integrated real-time data analysis was used for base calling (11). Default parameters were used except where otherwise noted. Quality assessment was performed with the use of the prinseq-lite program (v.0.20.4) (http://prinseq.sourceforge.net) (12). The total number of reads was 15,084,680. The number of reads passing the quality check was 14,555,656 (96.49%). The total number of joined reads was 13,451,084 (92.41% of cleaned reads). Sequences from Illumina sequencing were joined using the FLASH program (v.1.2.11) (13), loaded into Geneious Prime software (v.2020.2.4) (14), and trimmed with the BBDuk plugin (v.1.0) (https://sourceforge.net/projects/bbmap). Adapters on the right end and low-quality ends (quality score below 20%) were trimmed. Reads shorter than 200 bp were discarded. The reads were then subjected to preprocessing (https://www.geneious.com/tutorials/map-to-reference). The genome was assembled by using the “Map to reference” tool. The reads spanned the junction between the two linearized ends and overlapped with them, thus visually confirming a circular genome. As a reference, the BCG-I genome with accession number NZ_CP013741 was used with the Geneious mapper. A consensus sequence from aligned reads was extracted.

A second run of sequencing to generate long reads for scaffolding was performed with PromethION technology (Oxford Nanopore Technologies, Oxford, UK). The DNA ends were prepared for adapter attachment with the Nanopore NEBNext FFPE DNA repair mix. Barcodes were added with the Nanopore EXP-NBD104 kit. No fragment size selection or shearing was applied. The library was constructed using the Nanopore SQK-LSK109 ligation kit, and sequencing was performed with a PromethION instrument (PromethION flow cells, FLO-PRO002; Oxford Nanopore Technologies).

For base calling, the original Nanopore signal was processed with Guppy software integrated in the Nanopore software (v. 3.03). Quality control (QC) was performed with the online NanoPlot service (http://nanoplot.bioinf.be). The QC showed that the total number of bases was 123,155,602; the number of reads was 139,414, the median read length was 604 bp, the read *N*_50_ value was 1,178 bp, the median read quality score was 11, and the number (percentage) of reads with a quality score of >7 was 133,439 (95.7%). Nanopore reads were subjected to a similar procedure for reference mapping in Geneious Prime but without preprocessing steps.

The complete genome sequence of BCG SL222 Sofia comprised one circular chromosome consisting of 4,370,706 bp, with a GC content of 65.60%. Annotation was generated with the NCBI Prokaryotic Genome Annotation Pipeline (PGAP) (v.4.13) (15). A total of 4,078 genes were predicted. Four high-quality single-nucleotide polymorphisms were identified between BCG SL222 Sofia and BCG-I generation 368.

### Data availability.

The annotated genome sequence of BCG SL222 Sofia was deposited under GenBank accession number CP064405. The accession number for the Illumina raw reads is SRX9465445, and that for the Nanopore raw reads is SRX9523320.
